# A Single-center experience of subperineural resection of intracranial schwannomas and the clinical course following subtotal resection

**DOI:** 10.3389/fonc.2026.1830390

**Published:** 2026-06-24

**Authors:** Azuna Tomioka, Ryota Tamura, Junki Sogano, Kosuke Karatsu, Konosuke Ishikawa, Taichi Sayanagi, Takenori Akiyama, Ryo Ueda, Masahiro Toda

**Affiliations:** Department of Neurosurgery, Keio University School of Medicine, Shinjuku-ku, Tokyo, Japan

**Keywords:** non-vestibular tumor, recurrence, schwannoma, subperineural resection, subtotal resection, vestibular tumor

## Abstract

**Background:**

Subperineural resection technique is regarded as an effective surgical strategy for preserving neurological function in vestibular schwannomas (VSs); however, its application in non-vestibular schwannomas (NVSs) has been rarely reported. Furthermore, total resection (TR) cannot always be achieved, as dissection along the appropriate plane may be challenging, occasionally resulting in subtotal removal (STR). We have consistently employed the subperineural resection technique for all intracranial schwannomas. In this study, we aimed to evaluate its effectiveness in preserving neurological function and analyzed postoperative outcomes, with particular emphasis on the clinical course following subtotal resection (STR).

**Methods:**

186 patients of intracranial schwannomas were retrospectively analyzed, focusing on the extent of resection and postoperative behavior of residual tumors. Tumor origin by cranial nerve (CN) was as follows: CN II (n=2), CN IV (n=1), CN V (n=15), CN VI (n=1), CN VII (n=4), CN VIII (n=148), CN IX–XI (n=12), and CN XII (n=3).

**Results:**

With subperineural resection, TR rates for CN V, VII, VIII, IX–XI, and XII schwannomas were 53.3%, 75%, 70.3%, 50%, and 0%, respectively. Among STR cases, regrowth occurred in 12/44 CN VIII (27.3%) and 4/7 CN V (57.1%), while retreatment was required in 4/44 and 0/7, respectively. For CN VIII schwannomas, postoperative planimetric and volumetric growth rates were 0.22 mm/year and 126.3 mm³/year; corresponding values for CN V were 0.7 mm/year and 23 mm³/year. In contrast, regrowth occurred in 5/6 CN IX–XI (83.3%) and 2/3CN XII (66.7%), with retreatment required in 2/6 CN IX–XI and 1/3 CN XII, indicating a relatively higher proportion despite the small sample size.

**Conclusion:**

Subperineural resection is an effective strategy, however, STR remains unavoidable in some cases. In CN VIII and CN V schwannomas, retreatment rates were low relative to regrowth. The optimal timing of postoperative radiotherapy following STR remains controversial, and further large-scale analyses stratified by the nerve of origin are warranted.

## Introduction

Schwannomas are tumors arising from Schwann cells of the peripheral nerves (1). Most are located on cranial nerve (CN) VIII, but they can develop along any peripheral nerve (2–5).

In schwannoma surgery, subperineural resection is necessary to achieve satisfactory result (6, 7). When total resection (TR) cannot be achieved, management options include stereotactic radiosurgery, reoperation, or close observation; however, optimal treatment strategies for residual tumors remain controversial in the absence of established guidelines. Even for incompletely resected vestibular schwannomas (VSs), available data are limited (8–12), and evidence regarding residual non-vestibular schwannomas (NVSs) is even more scarce (13–16). Recently, schwannomas have been increasingly recognized to exhibit distinct genetic backgrounds depending on their nerve of origin, drawing attention to potential differences in their biological behavior. Intracranial schwannomas demonstrate location-dependent genotypic and phenotypic heterogeneity, with identical histology yielding divergent clinical courses (17, 18). Recent studies comparing vestibular (VS) and non-vestibular schwannomas (NVSs) demonstrate nerve-specific genetic landscapes (19, 20) However, surgical outcomes across cranial nerve origins remain poorly characterized. Therefore, in this study, we retrospectively analyzed surgically treated VSs and NVSs at our institution to evaluate the effectiveness of the subperineural resection technique and to characterize the postoperative course of residual tumors after STR across different cranial nerve origins.

## Methods

### Patient population

We reviewed patients who underwent intracranial schwannoma resection in our institution from April 2011 to March 2022. Subperineural resection was attempted in all cases. Patients with NF2-related schwannomatosis (21) and patients with prior resections or inadequate follow-up were excluded. Institutional review board approval was obtained (reference number: 20050002). Written informed consent was obtained from all participants for use of their data for research purposes.

### Data collection

Clinical and surgical data were obtained from the medical record. Tumor characteristics and tumor volume were evaluated using pre- and postoperative magnetic resonance imaging with and without contrast. Tumor size was assessed based on its maximum diameter. A volume segmentation method on the basis of gadolinium enhanced T1-weighted images or T2-weighted images was performed (22, 23). The extent of resection was determined based on intraoperative findings and postoperative imaging, and was classified as either gross total resection (GTR) or subtotal resection (STR). VSs were classified using the Koos classification (24).

Postoperative symptoms were evaluated at last follow-up. Facial nerve function was assessed using the House–Brackmann scale, and auditory function using the American Academy of Otolaryngology–Head and Neck Surgery hearing classification (25, 26). Facial nerve function grades I and II and auditory function grades A and B were considered to indicate functional preservation (25, 26). Swallowing function was assessed based on the ability to consume a free diet.

According to the San Francisco criteria, tumor regrowth during follow-up was defined as a >2 mm linear increase in maximum diameter or a >20% increase in tumor volume compared with the initial postoperative imaging obtained 1 month after surgery. Follow-up time was calculated from the date of surgery to the date of the final imaging examination.

### Surgical methods

Surgery was performed by five neurosurgeons, with two primary operators; however, the fundamental principles and techniques were standardized across the institution. The craniotomy was chosen based on tumor location. Endonasal endoscopic and transcervical procedures were performed collaboratively with otolaryngologists, whereas all other surgeries were carried out solely by neurosurgeons. After exposing the tumor by opening the arachnoid fold, electrical stimulation identified the nerve. Internal debulking and subperineural dissection along the nerve-derived membrane were performed. Appropriate intraoperative monitoring, including facial nerve, ABR, lower cranial nerve, and other relevant modalities, was employed.

### Statistical analysis

Statistical analyses were performed using JMP Pro 17 software (SAS Institute Inc., Cary, NC, USA). Categorical data were compared using the chi-square or Fisher’s exact test as appropriate. Continuous data were compared using the Mann–Whitney U test. Kaplan–Meier curves were generated to assess regrowth-free survival by nerve of origin, and group differences were evaluated using the log-rank test. A p value <0.05 was considered statistically significant.

## Results

### Patient and tumor characteristics

A total of 186 patients with intracranial schwannomas were analyzed. The anatomical tumor origin was CN II in two patients, CN IV in one, CN V in 15, CN VI in one, CN VII in four, CN VIII in 148, CN IX–XI in 12, and CN XII in three. Patient characteristics are shown in **Supplementary Table 1**. For schwannomas of CNs V, VII, VIII, IX–XI, CN XII, the TR rates were 53.3%, 75%, 70.3%, 50%, and 0%, respectively. Tumor location, surgical approach, and other information are summarized in **Table 1**.

**Table 1:** Postoperative course after subtotal removal in lower and hypoglossal nerve schwannomas.

Origin of tumor	Location of tumor	n	Approach	n	Extent of resection	n	Rate of TR (%)	Mean operation time (hour)	Mean intraoperative blood loss (ml)
II	Orbit	2	SOA	2	TR	2	100	4.2	57
IV	PCF	1	ATP	1	TR	1	100	6.8	87
V	Extracranial	1	EEA	1	STR	1	53.3	4.3	100
	MCF	5	FTA	1	STR	1		8.7	120
			STA	2	TR	1		6.9	115
					STR	1			
			ATP	2	STR	2		7.1	239
	MCF+PCF	7	ATP	6	TR	5		7.7	192
					STR	1			
			LSA	1	STR	1		5.3	250
	MCF+Extracranial	1	ZIA	1	TR	1		4.4	418
	MCF+Extracranial+PCF	1	STA	1	TR	1		5.4	190
VI	Extracranial+PCF	1	ATP	1	STR	1	0	6.9	91
VII	MCF+PCF	1	STA	1	TR	1	75	6.5	115
	PCF	1	LSA	1	TR	1		5.2	110
	IAC+CPA	2	LSA	2	TR	1		4.5	30
					STR	1			
LCN	PCF	6	LSA	6	TR	4	50	7.7	240
	PCF+Extracranial	6	LSA	3	STR	3		5.7	138
			LSA+TJA	3	TR	2		11.6	325
					STR	1			
XII	PCF+Extracranial	3	TCA	1	STR	1	0	6.2	80
			TCEA	2	STR	2		5.8	113

A total of 186 patients with intracranial schwannomas are shown. Number of each cases, Tumor location, surgical approach, extent of resection, rate of total resection, operation time, and intraoperative blood loss are summarized. ATP, anterior transpetrosal approach; CPA, cerebellopontine angle; EEA, endonasal endoscopic approach; FTA, frontotemporal approach; TR, total resection; IAC, internal auditory canal; LCA, lateral suboccipital approach; MCF, middle cranial fossa; MFA, middle fossa approach; PCF, posterior cranial fossa; SOA, supraorbital approach; STA, subtemporal approach; TCA, transcondylar approach; TCEA, transcervical approach; ZIA, zygomatic infratemporal approach.

### Symptom improvement after surgery

Mean follow-up for schwannomas of CN II, IV, V, VI, VII, VIII, IX–XI, and XII was 3.6, 1, 4.8, 5.5, 5.5, 4.5, 4.3, and 7.3 years, respectively. Postoperative outcomes of preoperative neurological symptoms are shown in **Table 2**. In CN V schwannomas (n=15), facial hypesthesia was present in eight patients and trigeminal neuralgia in two; postoperative improvement was observed in six and two patients, respectively. Among CNs IX–XI schwannomas (n=12), three patients (25%) had dysphagia, with improvement in one case. No neurological improvement was observed in CN XII schwannomas (n=3).

**Table 2:** Functional improvement of preoperative symptoms after the operation.

Origin of tumor	Preoperative symptoms (n, %)	Functional improvement after operation (n, %)	Duration until improvement (month)
II	Exophthalmos (2, 100)	2, 100	0.1
	Diplopia (1,50)	1, 100	8
	Visual acuity disturbance (1, 50)	0, 0	–
IV	Dizziness (1, 100)	1, 100	2
V	Facial hypoesthesia (8, 53.5)	6, 75	8.1
	Facial numbness (4, 26.7)	1, 25	12
	Trigeminal neuralgia (2, 13.3)	2, 100	13
	Dizziness (2, 13.3)	2, 100	5
	Trigeminal motor impairment (1, 6.7)	0, 0	–
	Diplopia; CN VI paresis without diplopia (1, 6.7; 1, 6.7)	1, 100; 1, 100	4; 1
	Hearing disturbance (1, 6.7)	1, 100	1
VI	Diplopia (1, 100)	1, 100	1
VII	Facial paralysis (2, 50)	1, 50	1
	Hearing disturbance (2, 50)	0, 0	–
	Dizziness (1, 25)	1, 100	1
	Facial spasm (1, 25)	1, 100	1
	Facial numbness (1, 25)	0, 0	–
VIII	Hearing disturbance (95, 64.2)	0, 0	–
	Dizziness (32, 21.6)	19, 59.3	1.9
	Tinnitus (19, 12.8)	6, 31.6	2.7
	Facial sensory disturbance (17, 11.5)	3, 17.6	1
	Facial paralysis (14, 9.6)	1,7.1	6
	Facial numbness (5, 3.4)	2, 40	1
	Facial pain (4, 2.7)	1, 25	1
LCN	Dizziness (6, 50)	6, 100	1.2
	Dysphagia (3, 25)	1, 33.3	37
	Hoarseness (2, 16.7)	0, 0	–
	Tongue deviation (2, 16.7)	0, 0	–
	Hearing disturbance (2, 16.7)	0, 0	–
	Facial numbness (1, 8.3)	1, 100	2.5
XII	Tongue deviation (3, 100)	0, 0	–
	Tongue atrophy (1, 33.3)	0, 0	–
	Hoarseness (1, 33.3)	0, 0	–

This shows the number and incidence of preoperative neurological symptoms according to type of schwannoma, as well as the frequency, proportion, and duration of postoperative symptom resolution. LCN, lower cranial nerve.

Postoperative improvement rates for each symptom are shown in **Supplementary Table 2**. Hearing loss showed minimal postoperative recovery regardless of schwannoma origin. Dizziness improved postoperatively in 59.3% of CN VIII schwannoma patients (19/32), compared with 100% of patients with NVS schwannomas (p = 0.02).

### New symptoms after surgery

New postoperative symptoms and recovery rates are summarized in **Table 3**. In CN VIII schwannomas, postoperative hearing loss occurred in 21 cases (14.2%), with no recovery observed. Facial weakness developed in 47 patients (31.8%), with improvement in 39 (83%). All received in-hospital facial nerve rehabilitation provided by physical and speech therapists. Most recovery occurred within 6 months postoperatively (**Supplementary Table 3**). In CN IX–XI schwannomas, postoperative dysphagia and hoarseness occurred in 66.7% and 40% of patients, respectively, with recovery in 83.3% of dysphagia cases and all cases of hoarseness.

**Table 3:** New-onset symptoms after the operation.

Origin of tumor	New onset postoperative symptom (n, %)		Functional improvement (n, %)	Duration until the improvement (month)
II	Ptosis	1, 50	1, 100	1
	Eye movement disorder	1, 50	1, 100	2
V	Hypoesthesia	3, 20	1, 33.3	4
	Abnormal occlusion	2, 13.3	0, 0	–
	Diplopia by CN IV palsy	1, 6.7	1, 100	6
	Diplopia by other nerves	1, 6.7	1, 100	9
	Facial paralysis	1, 6.7	1, 100	4
VII	Facial paralysis	2, 50	1, 50	3
VIII	Facial paralysis	47, 31.8	39, 83	4.6
	Hearing disturbance	21, 14.2	0, 0	–
	Deglutition disorder	2, 1.4	1, 50	5
	Facial sensory disturbance	1, 0.7	1, 100	0.5
	Diplopia	1, 0.7	1, 100	1
	Motor paralysis	1, 0.7	0, 0	–
	Consciousness disorder	1, 0.7	0, 0	–
LCN	Dysphagia	6, 66.7	5, 83.3	4
	Hoarseness	4, 40	4, 100	13
	Facial paralysis	2, 16.7	2, 100	3.8
	Diplopia by CN VI palsy	1, 8.3	1, 100	1
	Hearing disturbance	2, 20	1,50	1
	Tongue deviation	1, 10	1, 100	1
	dizziness	1, 16.7	1,100	1

The incidence of new postoperative symptoms and their rate of improvement during follow-up according to type of schwannoma are shown. LCN, lower cranial nerve.

### Extent of resection

Among CN VIII schwannomas, the totally resected CN VIII schwannomas were significantly smaller and less often Koos grade 4 than subtotally resected tumors (*p* < 0.01; **Table 4**). Overall hearing and facial nerve preservation rates were 69.0% and 94.1%, respectively. Facial nerve preservation was comparable between the TR and STR groups (92.9% vs. 96.9%, p = 0.72), as was hearing preservation (68.3% vs. 70.6%, p = 0.85). Recurrence occurred in 29.5% of subtotally resected tumors but in none of the totally resected tumors (p < 0.01).

**Table 4:** Results of VIII schwannoma after TR or STR.

CN VIII schwannoma		TR	STR	Total	P value
N (%)		104 (70.3)	44 (29.7)	148	–
Age (range)		53, 19-80	53.3, 16-84	53.1, 16-84	0.92
Sex	Male	48 (46.2)	15 (34.1)	63 (42.6)	0.56
	Female	56 (53.8)	29 (65.9)	85 (57.4)	
Tumor size at preoperation (mm, range)		21.3 (3-51)	27.7 (7-53)	23.2 (3-51)	<0.01
Koos grade	1	3 (2.9)	2 (4.5)	5 (3.4)	<0.01
	2	33 (31.7)	7 (15.9)	40 (27)	
	3	43 (41.3)	12 (27.3)	55 (37.2)	
	4	16 (15.4)	17 (38.6)	33 (22.3)	
	4OM	9 (8.7)	6 (13.6)	15 (10.1)	
Tumor characteristics	Solid	61 (58.7)	27 (61.4)	88 (59.5)	0.86
	Cystic	43 (41.3)	17 (38.6)	60 (40.5)	
Preservation rate of CN VII function (%)		92.9	96.9	94.1	0.72
Hearing Preservation (%)		68.3	70.6	69.0	0.85
Tumor regrowth (n, %)		0, 0	13, 29.5	13, 8.8	<0.01
Mean FU time (m, range)		53.4, 12-120	55.4, 12-122	54.0, 12-122	–
Improvement of CN VIII function at 1,3,5,7,9 years (n, %)	at 1year	104, 100	44, 100	148, 100	–
	at 3years	72, 69.2	30, 68.2	102, 68.9	
	at 5 years	53, 51.0	24, 54.5	77, 52.0	
	at 7 years	25, 24.0	12, 27.3	37, 25	
	at 9 years	6, 5.8	5, 11.4	11, 7.4	

This table presents the characteristics, dimensions, and postoperative course of tumors in 148 patients with CN VIII schwannomas. Data are further stratified into the STR group and the TR group. CN, cranial nerve; FU, follow upr; TR, total removal; STR, subtotal removal

The corresponding results for NVS schwannomas are summarized in **Supplementary Tables 4**-**7**. No recurrences were observed after total resection. In contrast, recurrence rates after subtotal resection were 57.1% for CN V, 100% for CN VII, 83.3% for CN IX–XI, and 66.7% for CN XII.

### Behavior of residual tumor

**Figure 1:**
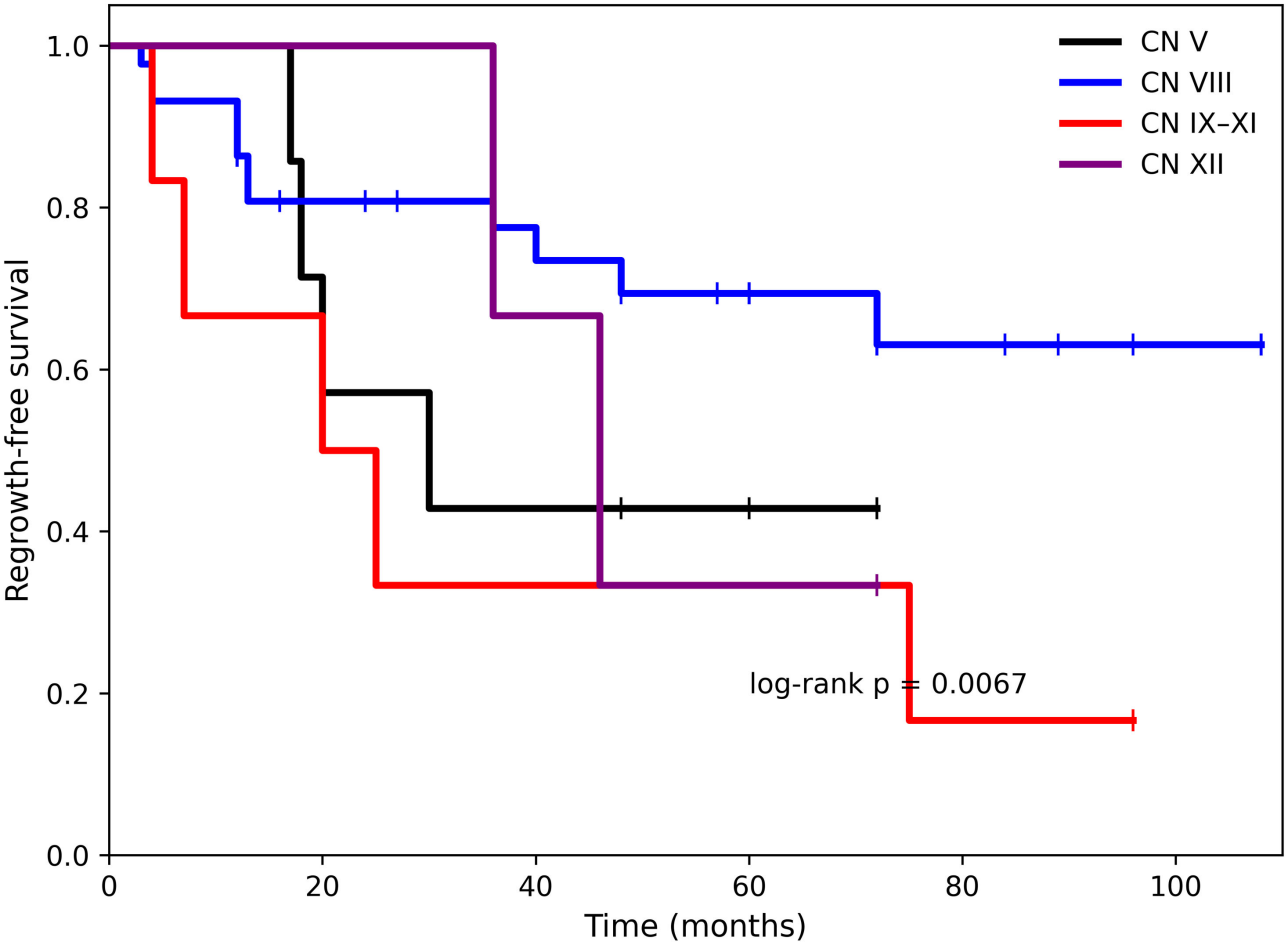
Kaplan–Meier curves by cranial nerve origin. Kaplan–Meier curves of regrowth-free survival after subtotal resection stratified by cranial nerve origin. Regrowth-free survival differed among subgroups, with CN VIII schwannomas demonstrating a more gradual decline and CN IX–XI schwannomas showing earlier decline. Tick marks indicate censored observations. Differences among groups were statistically significant (log-rank test, χ² = 10.01, p = 0.0067).

The size and behavior of residual tumors, regrowth rates, and re-treatment modalities in the STR group are presented in **Tables 5**, **6**, **7**. Tumor regrowth occurred in 12 of 44 CN VIII schwannomas (27.3%), with retreatment required in four cases (two reoperations, two radiosurgery). Regrowth was observed in four of seven CN V schwannomas, all remaining stable on follow-up. Five of six CN IX–XI schwannomas showed regrowth, with three requiring retreatments. Among four cases with complete intracranial but residual extracranial tumors, regrowth occurred in three. Two of three CN XII schwannomas regrew, and one required radiosurgery. Kaplan–Meier curves of regrowth-free survival stratified by cranial nerve origin are shown in **Figure 1**. Regrowth-free survival differed among subgroups, with CN VIII schwannomas demonstrating a more gradual decline and CN IX–XI schwannomas showing earlier decline. These differences were statistically significant (log-rank test, χ² = 10.01, p = 0.0067).

**Table 5:** Postoperative course after subtotal removal in trigeminal, facial, and vestibular nerve schwannomas.

Origin of tumor			Regrowth	Stable or reduction	P value
V	N, %		4, 57.1	3, 42.9	–
	Age (range)		51.5, 43-63	56, 33-79	0.67
	Sex (n, %)	Male	2, 50	1, 33.3	0.66
		Female	2, 50	2, 66.7	
	Tumor characteristics (n, %)	Solid	3, 75	0, 0	0.05
		Cystic	1, 25	3, 100	
	Size of residual tumor (mm, range)		14.3, 5-19	7.3, 5-11	0.09
	Volume of residual tumor (mm3)		533.9	517.0	0.45
	Speed of regrowth (mm/year)		0.7	–	–
	Mean regrowth free survival time (years)		1.8	–	–
VII	N, %		1, 100	0, 0	–
	Age (range)		59, -	–	–
	Sex (n, %)	Male	0, 0	–	–
		Female	1, 100	–	–
	Tumor characteristics (n, %)	Solid	0, 0	–	–
		Cystic	1, 100	–	–
	Size of residual tumor (mm, range)		13	–	–
	Volume of residual tumor (mm3)		870.2	–	–
	Speed of regrowth (mm/year)		0.7	–	–
	Mean regrowth free survival time (years)		1.8	–	–
VIII	N, %		12, 27.3	32, 72.7	–
	Age (range)		54.1, 24-66	52.9, 16-84	0.10
	Sex	Male	5, 41.7	13, 40.6	0.86
		Female	7, 58.3	19, 59.4	
	Tumor characteristics (n, %)	Solid	8, 66.7	19, 59.3	0.92
		Cystic	4, 33.3	13, 40.7	
	Location of residual tumor (n, %)	IAC	5, 41.7	7, 21.9	0.67
		IAC+CPA	7, 58.3	25, 78.1	
	Size of residual tumor (mm, range)		17.1, 6.8-38.6	14.2, 6.0-23.8	0.37
	Volume of residual tumor (mm3)		1422.3	1158.9	0.62
	Speed of regrowth (mm/year)		0.22	–	–
	Mean regrowth free survival time (year)		2.5	–	–

The behavior of residual tumors and retreatment rates for CN V, VII, VIII schwannoma are shown. The Cases in which subtotal removal was performed are divided into two groups --those in which residual tumors exhibited regrowth and those in which they remained stable or showed reduction. The number of patients, tumor characteristics, and sizes of the residual tumors between these groups are summarized. For the regrowth group, the rate of tumor regrowth is shown. CN, cranial nerve; CPA, cerebellopontine angle; IAC, internal auditory canal; STR, subtotal removal.

**Table 6:** Postoperative course after subtotal removal in lower and hypoglossal nerve schwannomas.

Origin of tumor			Regrowth	Stable or Reduction	P value
LCN	N, %		5, 83.3	1, 16.7	–
	Age (range)		51.8, 42-62	56, 56	0.37
	Sex (n, %)	Male	2, 40	1, 100	0.27
		Female	3, 60	0, 0	
	Tumor characteristics (n, %)	Solid	2, 40	0, 0	0.67
		Cystic	3, 60	1, 100	
	Size of residual tumor (mm, range)		17.8, 9-27	11, 11	0.17
	Volume of residual tumor (mm3)		1883.1	950.2	0.66
	Location of residual tumor (n, %)	Intracranial	2, 40	0, 0	0.43
		Intra+extracranial	0, 0	0, 0	
		Extracranial	3, 60	1, 100	
	Speed of regrowth (mm/year)		3.87	–	–
	Mean regrowth free survival time (year)		2.4	–	–
XII	N, %		2, 66.7	1, 33.3	–
	Age (range)		41.5, 38.45	70, 70	0.13
	Sex (n, %)	Male	1, 50	1, 100	0.39
		Female	1, 50	0, 0	
	Tumor characteristics (n, %)	Solid	2, 100	1, 100	–
		Cystic	0, 0	0, 0	
	Size of residual tumor (mm, range)		22.5, 18,27	17, 17	0.44
	Volume of residual tumor (mm3)		9057.9	2712.8	0.33
	Location of residual tumor (n, %)	Intracranial	0, 0	0, 0	–
		Intra+extracranial	2, 100	1, 100	
	Speed of regrowth (mm/year)		1.72	–	–
	Mean regrowth free survival time (year)		3.4	–	–

The behavior of residual tumors and retreatment rates for LCN and CN XII schwannoma are shown. The Cases in which subtotal removal was performed are divided into two groups --those in which residual tumors exhibited regrowth and those in which they remained stable or showed reduction. The number of patients, tumor characteristics, and sizes of the residual tumors between these groups are summarized. For the regrowth group, the rate of tumor regrowth is shown.CN, cranial nerve; LCN, lower cranial nerve; STR, subtotal removal

**Table 7:** Postoperative Tumor Growth Rates and Cases Requiring Re-treatment in the STR Group Originating from Various Cranial Nerves.

Origin of tumor	STR (n)	Regrowth (n, %)	Mean postoperative planimetric growth rate after STR(mm/year)	Mean postoperative volumetric growth rate after STR(mm3/year)	Mean preoperative growth rate (mm/year)	Postoperative treatment (n)
V	7	4, 57.1	0.7	23.0	3.4	–
VIII	44	12, 27.3	0.2	126.3	3.5	Reoperation: 2 Cyberknife: 1 Gamma knife: 1
LCN	6	5, 83.3	3.9 (median 2.2mm/year, IQR 1.8-5.6)	5050.1 (median: 3274.5mm³/year, IQR 360.1–6099.6)	3.7	Reoperation: 2 Gamma knife: 1
XII	3	2, 66.7	1.7 (2.17, 1.27mm/year)	1021.3 (1219.8, 822.8 mm³/year)	–	Cyberknife: 1

This table summarizes postoperative tumor growth rates, as well as the number and characteristics of cases requiring re-treatment, among patients who underwent subtotal resection (STR). The mean preoperative tumor growth rates in patients who were initially managed with observation after presentation are also shown. LCN, lower cranial nerve; STR, subtotal removal

Postoperative growth rates of residual tumors after STR are summarized in **Table 7**. In CN VIII schwannomas, the mean planimetric and volumetric growth rates were 0.22 mm/year and 126.3 mm³/year, respectively. The corresponding rates for CN V schwannomas were 0.7 mm/year and 23.0 mm³/year; for CN IX–XI schwannomas, 3.87 mm/year whereas the median was 2.2 mm/year (IQR 1.8-5.6) and 5051 mm³/year the median: 3274.5 mm³/year (IQR 360.1–6099.6); and for CN XII schwannomas, 1.72 mm/year (2.17mm/year, 1.27mm/year) and 1021.3mm³/year (1219.8mm³/year, 822.8 mm³/year).

## Discussion

### Pre-and postoperative symptoms, the effectiveness of the subperineural resection technique

Preservation of nerve function is the primary concern of schwannoma surgery. The importance of subperineural dissection has been well recognized (6, 27), as it minimizes neural disturbance and serves as a reliable anatomical landmark. However, resection strategies remain variable, and outcome data on true subperineural dissection—particularly for NVSs—are limited. We therefore report our institutional experience.

Our rates of hearing preservation (69%) and facial function preservation (94.1%) in VSs are better than those in most previous reports (28), likely due to strict adherence to the subperineural resection technique. Postoperative facial function tended to gradually improve over time, regardless of tumor size, and most recovery occurred within the first 6 months. It may be reasonable to consider facial nerve reconstruction 6 to 12 months after surgery in patients with persistent severe weakness or paralysis.

Reported postoperative improvement rates for trigeminal nerve-related symptoms in CN V schwannomas are as follows: 16%–59% for facial hypoesthesia, 17%–35% for facial numbness, 91%–100% for trigeminal neuralgia, and 21%–33% for trigeminal motor function (29–31). In contrast with facial numbness, trigeminal neuralgia frequently improves rapidly after surgery (31). Our improvement rates were 50% for facial numbness and 100% for trigeminal neuralgia, which are in line with those in previous studies.

IN CN IX–XI schwannomas, preoperative symptoms tended to show poor postoperative improvement, whereas new-onset postoperative symptoms were more likely to improve in our study, consistent with previous reports (32–34). Surgical approaches for CN IX–XI schwannomas vary across centers, with postoperative lower cranial nerve deficits reported in 6%–100%, facial nerve weakness in 11%–80%, and hearing loss in 4%–45% (32–34). In a 2016 study, approximately 60% of jugular foramen schwannoma patients developed new-onset dysphagia, with over half improving (34). In our study, 88.3% with dysphagia and all with hoarseness recovered, indicating favorable outcomes.

IN CN XII schwannomas, symptoms vary based on tumor location; however, hypoglossal nerve dysfunction has been reported in approximately 80% of patients (13, 35). Moreover, patients with pre-existing hypoglossal nerve dysfunction are unlikely to improve postoperatively (13, 35). None of our cases showed symptom improvement. However, no new neurological deficits were noted.

In summary, subperineural removal may be recommended not only for VS resection, but also for resection of NVSs to preserve and even improve nerve function. Although we attempt to apply this technique in all cases, the perineurial plane is not always clearly identifiable, particularly in large or cystic tumors. Nevertheless, we endeavor to preserve the perineurium as much as possible, especially in regions where nerve fibers are densely concentrated and closely associated with the tumor, such as around neural foramina. Even when this approach results in subtotal resection or incomplete functional preservation, our findings suggest that efforts to maintain the perineurial layer may still confer functional benefit.

### Extent of resection and postoperative course of residual tumor

At our institution, TR was generally attempted, with its extent limited by nerve adhesion and intraoperative neuromonitoring changes. Although TR has been reported as an independent risk factor for postoperative facial weakness (30–50%) in CNVIII schwannomas (10, 36, 37), no statistically significant differences were observed between TR and STR groups in our series, despite slightly better outcomes in the STR group (facial function: 92.9% vs. 96.9%, p = 0.72; hearing: 68.3% vs. 70.6%, p = 0.85). Furthermore, regardless of the nerve of origin, no significant differences in pre- and postoperative nerve function were observed between the TR and STR groups. These findings suggest that an appropriate balance between maximal tumor removal and functional preservation was achieved. However, the potential influence of confounding factors, including tumor size and complexity, should be considered. Taken together, our results indicate that, with careful case selection and tailored surgical strategy, an appropriate extent of resection can be achieved while maintaining favorable functional outcomes.

An important clinical consideration is the optimal management strategy following STR. Previous studies indicate that 7-32% of residual VS tumors exhibit significant regrowth, with recurrence being 11 times more likely after STR (10, 11, 36, 37). In our study, regrowth occurred in 27.3% of STR cases but in none of the TR cases (p < 0.01). However, this finding should be interpreted with caution, as tumors in the STR group were significantly larger and more advanced at baseline, and dichotomization into GTR and STR may have introduced heterogeneity within the STR group, particularly as near-total resection was not analyzed separately.

The optimal management of residual tumors remains controversial, with increasing emphasis on early adjuvant radiotherapy (38, 39). The 2025 International Stereotactic Radiosurgery Society (ISRS) practice guidelines recommend a planned hybrid strategy consisting of STR followed by early stereotactic radiosurgery (SRS) to the residual tumor (40). In addition, the V-REX randomized clinical trial demonstrated that upfront SRS significantly reduced tumor volume compared with a wait-and-scan approach in patients with small- to medium-sized VSs (<2 cm) (41). However, residual tumors may exhibit biological behavior distinct from untreated tumors, and caution is warranted when directly extrapolating these findings to residual disease. Our results suggest that not all residual tumors require immediate intervention. In particular, among CN VIII and CN V schwannomas, a considerable proportion of STR cases may be suitable for initial observation. In our study, 27.3% of residual tumors regrew, most commonly 3–4 years after surgery, yet only a subset ultimately required retreatment. Among 44 CN VIII schwannomas treated with STR, 12 exhibited regrowth, whereas only 4 required additional intervention. Moreover, in cases without regrowth, the mean residual tumor volume was 1158.9 mm³. Some studies suggest that even partial resection may effectively control tumor growth, particularly when the residual tumor is small (42). In addition, prior studies have identified residual tumor diameter >15 mm or volume >400 mm³ as risk factors for regrowth, with volumes of 1000–2500 mm³ carrying particularly high-risk during observation (43–45). Accordingly, some institutions advocate early postoperative radiotherapy for remnants ≥ 1000 mm³. In contrast, our data suggest that residual tumors do not necessarily mandate immediate intervention, even in cases with relatively large residual volumes.

Growth rate analysis provides additional context for this perspective. Rosenberg et al. reported a mean growth rate of 0.90 mm/year for untreated VSs, compared with 0.35 mm/year for residual tumors (46). Syed et al. (36) reported 0.77 mm/year. In our series, the residual tumor growth rates were 0.22 mm/year and 126.3 mm³/year, markedly slower than the mean preoperative growth rate (3.53 mm/year) observed in patients initially managed with observation. This reduction in growth may be partly attributed to surgery-related devascularization. Oishi et al. described a ‘dura-like membrane’ surrounding CN VIII schwannomas originating from the dura of the internal auditory canal, suggesting that tumor growth may be dural vascular supply; thus, dural coagulation may contribute to postoperative growth control (47).

A similar trend was observed in CN V schwannomas. Although recurrence rates after STR have been reported to vary widely (11–100%) (14, 29, 48), some studies describe relatively indolent behavior following STR, with no need for reoperation over a mean follow-up of 4.3 years (28). In our series, regrowth occurred in 4 of 7 STR cases; however, none required additional treatment, with mean growth rates of 0.7 mm/year and 23.0 mm³/year. These findings suggest that careful observation may be a feasible postoperative strategy in selected cases.

In contrast, CN IX–XI and CN XII schwannomas may demonstrate a more aggressive postoperative course, with relatively rapid growth of residual tumors and a higher likelihood for retreatment. In one study, solid tumor, STR, and pathologic mitosis were identified as independent risk factors for recurrence and regrowth in jugular foramen schwannomas; with an approximately fivefold increased risk after STR (16). Other factors, such as a high MIB-1 index and jugular pocket extension, have also been associated with regrowth (12). For CN XII schwannomas, tumor regrowth was reported in six of 15 subtotally resected cases by Bindal et al. and in 50% cases by Nonaka et al., requiring reoperation (13, 49). In our series, two recurrent CN XII schwannomas were classified as type B intra-extracranial tumors (13). And similar to CN IX–XI schwannomas, the intracranial portion was completely resected, whereas the pharyngeal component was partially resected. Residual tumors in CN IX–XI and CN XII schwannomas demonstrated relatively high growth rates. In CN IX–XI schwannomas, the mean planimetric and volumetric growth rates were 3.87 mm/year and 5050.1 mm³/year, respectively, whereas the median values were 2.2 mm/year (IQR 1.8–5.6) and 3274.5 mm³/year (IQR 360.1–6099.6). In CN XII schwannomas, the mean planimetric and volumetric growth rates were 1.72mm/year and 1021.3mm³/year, with individual values of 2.17 and 1.27 mm/year, and 1219.8 and 822.8 mm³/year, respectively. A substantial proportion of cases required retreatment. These findings suggest that observation alone may not always be sufficient for CN IX–XI and CN XII schwannomas, in contrast to CN V and CN VIII schwannomas. Possible contributing factors include larger extracranial residual tumor volume and incomplete devascularization. In addition, tumor behavior is known to differ according to the nerve of origin. Trigeminal schwannomas are often considered relatively indolent, whereas schwannomas arising from the lower cranial nerves may exhibit more variable or potentially more aggressive growth characteristics. Emerging evidence further suggests that the molecular and genetic backgrounds of schwannomas may differ according to their nerve of origin (19, 20), which may partly explain the heterogeneity observed in our cohort.

However, these findings should be interpreted with caution. The small sample size in several subgroups limits the reliability of mean-based estimates, which are susceptible to distortion by outliers. Indeed, the substantial variability observed in median and interquartile ranges underscores this limitation. Given these considerations, the growth estimates presented here should be regarded as exploratory rather than definitive. Further studies with larger cohorts are needed to better characterize growth dynamics across different cranial nerve origins.

Additionally, regrowth was defined according to the San Francisco criteria (>2 mm linear increase or >20% volume increase) in this study. While this definition provides a standardized measure, it has inherent limitations: in particular, applying a strict 20% volume increase threshold to large STR remnants may underestimate clinically meaningful progression, as it can correspond to a substantial absolute increase in tumor burden and mass effect.

At our institution, routine postoperative imaging is generally performed every 6–12 months. However, this schedule is not strictly fixed and may be adapted according to individual risk factors. This approach is broadly consistent with commonly adopted postoperative surveillance strategies in the literature, where MRI is typically performed at 6 and 12 months followed by annual imaging thereafter (50). It is also conceptually aligned with recent consensus recommendations from the British Skull Base Society (BSBS, 2025), which advocate a risk-adapted, time-tapered surveillance strategy rather than fixed imaging intervals (51). Although early adjuvant radiotherapy may be considered after STR—particularly in cases with large residual tumors, including in our own practice—we generally favor an initial strategy of close surveillance, often with shorter imaging intervals in higher-risk cases. This approach is supported by the observation that many residual tumors—especially CN V and CN VIII schwannomas—remain stable, and that even when regrowth occurs, it is often transient and does not necessitate immediate intervention. Accordingly, additional treatment is reserved for cases with symptomatic progression, significant brainstem or cerebellar compression, or rapid radiological growth. In this context, our findings support a surveillance-first strategy in selected patients. In contrast, CN IX–XII schwannomas demonstrated a higher propensity for regrowth, suggesting that adjunctive therapy may need to be considered more proactively in these cases. While increasing emphasis has been placed on radiotherapy, it carries a small but non-negligible risk of facial nerve palsy and other neurological deficits, as well as potential complications such as radiation-induced edema and adhesions that may complicate subsequent reoperation. Accordingly, the choice between observation after STR and early radiotherapy should be individualized, taking into account multiple factors including patient age, tumor characteristics, and anatomical location. Kaplan–Meier analysis of tumor regrowth after STR revealed differences in regrowth-free survival among subgroups, indicating time-dependent and nerve-specific growth patterns. CN VIII schwannomas demonstrated a more gradual decline in regrowth-free survival, whereas schwannomas arising from the lower cranial nerves (CN IX–XI) tended to exhibit earlier regrowth. These findings indicate that postoperative tumor behavior after subtotal resection is not uniform but may vary depending on the cranial nerve of origin, with potential implications for postoperative surveillance strategies. However, these results should be interpreted with caution due to the limited sample size in several subgroups.

Finally, as tumor regrowth was observed even more than 10 years after surgery, long-term postoperative surveillance beyond a decade appears warranted. The BSBS emphasizes that postoperative remnants—especially those with larger residual size or nodular enhancement—may require prolonged follow-up. While surveillance of conservatively managed tumors may be discontinued after approximately 8.5 years, postoperative remnants may necessitate substantially extended follow-up, in some cases beyond 10–15 years (51). Therefore, we consider extended follow-up to be appropriate in our institutional practice.

## Limitations

The main limitations of this study include the relatively small sample size and limited follow-up duration, particularly in the non-vestibular schwannoma groups. Further subclassification or analysis using residual tumor volume was not feasible due to the limited sample size. In addition, exclusion of NF2 cases was based solely on clinical and radiological evaluation without genetic confirmation, and undiagnosed hereditary NF2 cannot be completely excluded. Larger studies with longer follow-up are warranted to validate these findings.

## Conclusion

Subperineural resection may facilitate functional preservation not only in vestibular schwannomas but also in non-vestibular schwannomas. Nevertheless, the perineurial layer cannot always be preserved, and some cases result in subtotal resection. In these cases, tumor growth may proceed more slowly than anticipated preoperatively. Therefore, postoperative management should be determined by a comprehensive assessment, taking into account histopathological features, the nerve of origin, and the expected growth dynamics.

## Data Availability

The original contributions presented in the study are included in the article/**Supplementary Material**. Further inquiries can be directed to the corresponding author.

## Supplementary material

Supplementary Table 1 Tumor characteristics originated from various intracranial nerves.

Supplementary Table 2 The relationship between representative preoperative symptoms and origin of tumors.

Supplementary Table 3 Improvement of new onset postoperative facial function in vestibular nerve schwannomas.

Supplementary Table 4 Extent of resection and clinical outcome in trigeminal nerve schwannomas.

Supplementary Table 5 Extent of resection and clinical outcome in facial nerve schwannomas.

Supplementary Table 6 Extent of resection and clinical outcome in lower cranial nerve schwannomas.

Supplementary Table 7 Extent of resection and clinical outcome in hypoglossal nerve schwannomas. This table presents the patient and tumor characteristics, and postoperative course of tumors in patients with CNXII schwannomas. Data are stratified into the STR group and the TR group. CN, cranial nerve; FU, follow up; TR, total removal; STR, subtotal removal.
