# Supplementary material for: A Single-center experience of subperineural resection of intracranial schwannomas and the clinical course following subtotal resection
Source: Front Oncol. 2026 Jun 24;16:1830390. doi: 10.3389/fonc.2026.1830390 (PMC13341456; doi:10.3389/fonc.2026.1830390)
Supplement: Supplementary Table 1 — Tumor characteristics originated from various intracranial nerves. [file DataSheet1.pdf]

Supplementary Appendix

Tomioka A, et al.

The following document contains all of the supplementary information and data for the manuscript, “ A Single-Center Experience of Subperineural Resection of Intracranial Schwannomas and the Clinical Course Following Subtotal Resection.”

Table of Contents

1) Table S1- Tumor characteristics originated from various intracranial nerves -----------------------------2

2) Table S2- The relationship between representative preoperative symptoms and origin of tumors --------3

3) Table S3- Improvement of new onset postoperative facial function in vestibular nerve schwannomas ---4

4) Table S4- Extent of resection and clinical outcome in trigeminal nerve schwannomas --------------------5

5) Table S5- Extent of resection and clinical outcome in facial nerve schwannomas -------------------------6

6) Table S6- Extent of resection and clinical outcome in lower cranial nerve schwannomas -----------------7

7) Table S7- Extent of resection and clinical outcome in hypoglossal nerve schwannomas -------------------8

Origin of tumors	Number (%)	Age (range)	Sex (Male: Female)
**II**	2 (1.1)	55.5 (53-58)	0:2
**IV**	1 (0.5)	80	1:0
**V**	15 (7.6)	46.9 (19-72)	5:10
**VI**	1 (0.5)	47	1:0
**VII**	4 (2.2)	53.2 (32-74)	1:3
**VIII**	148 (80.0)	52.8 (16-84)	63:85
**LCN**	12 (6.5)	43.5 (16-62)	7:5
**XII**	3 (1.6)	51 (38-70)	2:1
**Total**	186	53.6 (16-84)	80:106

Supplemental Table 1: Tumor characteristics originated from various intracranial nerves

The patient characteristics of the 186 patients with intracranial schwannomas who underwent surgery at our institution are shown.

LCN, lower cranial nerve

Symptom	Origin of tumors	Case with preoperative symptom (n)	Case with improvement in a symptom after the operation (n, %)	Duration until improvement in a preoperative symptom (month)
**Dizziness**	IV	1	1, 100	2
	V	2	2, 100	5
	VII	1	1, 100	1
	VIII	32	19, 59.3	1.9
	LCN	6	6, 100	1.2
**Facial paralysis**	VII	2	1, 50	3
	VIII	14	1, 7.1	6
**Hearing disturbance**	V	1	1, 100	1
	VII	2	0, 0	-
	VIII	95	0, 0	-
	LCN	2	0, 0	-

Supplemental Table 2: The relationship between representative preoperative symptoms and origin of tumors

This table shows the commonly observed preoperative symptoms categorized by tumor origin and this indicates both the improvement rates of these symptoms and the time required for their resolution.

LCN, lower cranial nerve

Koos classification	3 days after the operation (%)	At the time of discharge (%)	3 months after discharge (%)	6 months after discharge (%)	1 year after discharge (%)
**1**	100	100	100	100	100
**2**	88.9	88.9	92.6	100	100
**3**	81.1	86.5	91.9	97.30	97.3
**4**	66.7	73.3	83.3	90	100
**4OM**	50	50	60	80	80
**Total**	77	79.8	85.6	93.4	94.1

Supplemental Table 3: Improvement of new onset postoperative facial function in vestibular nerve schwannomas

This Table shows the temporal progression of symptom improvement in patients who developed new postoperative facial nerve palsy in CN VIII schwannomas.

CN, cranial nerve; LCN, lower cranial nerve OM,over midline

<table>
<tr><th colspan="2">V schwannoma</th><th>TR group</th><th>STR group</th><th>Total</th><th>P value</th></tr>
<tr><td colspan="2">N, %</td><td>8, 53.3</td><td>7, 46.7</td><td>15, 100</td><td>-</td></tr>
<tr><td colspan="2">Age, range</td><td>40.9, 19-72</td><td>53.4, 33-79</td><td>46.7, 19-79</td><td>0.18</td></tr>
<tr><td rowspan="2">Sex (n, %)</td><td>Male</td><td>2, 25</td><td>3, 42.9</td><td>5, 33.3</td><td rowspan="2">0.60</td></tr>
<tr><td>Female</td><td>6, 75</td><td>4, 57.1</td><td>10, 66.7</td></tr>
<tr><td colspan="2">Tumor size at preoperation (mm, range)</td><td>33.4, 25-43</td><td>28.4, 18-49</td><td>31.1, 18-49</td><td>0.34</td></tr>
<tr><td rowspan="2">Tumor characteristics (n, %)</td><td>Solid</td><td>5, 62.5</td><td>3, 42.9</td><td>8, 53.3</td><td rowspan="2">0.45</td></tr>
<tr><td>Cystic</td><td>3, 37.5</td><td>4, 57.1</td><td>7, 46.7</td></tr>
<tr><td rowspan="2">Change of preoperative CN V function after the operation (n, %)</td><td>Stabilized</td><td>6, 75</td><td>6, 85.7</td><td>12, 80</td><td rowspan="2">0.60</td></tr>
<tr><td>Permanent deterioration</td><td>2, 25</td><td>1, 14.3</td><td>3,20</td></tr>
<tr><td colspan="2">Tumor regrowth (n, %)</td><td>0, 0</td><td>4, 57.1</td><td>4, 26.7</td><td>0.03</td></tr>
<tr><td colspan="2">Mean FU time (month, range)</td><td>38.4, 13-72</td><td>79.2, 48-120</td><td>57.4, 13-120</td><td>-</td></tr>
<tr><td rowspan="5">Improvement of CN V function at 1,3,5,7,9 years (n, %)</td><td>at 1year</td><td>8, 100</td><td>7, 100</td><td>15, 100</td><td rowspan="5">-</td></tr>
<tr><td>at 3years</td><td>4, 50</td><td>7, 100</td><td>11, 73.3</td></tr>
<tr><td>at 5 years</td><td>2, 25</td><td>6, 85.7</td><td>8, 53.3</td></tr>
<tr><td>at 7 years</td><td>0, 0</td><td>2, 28.6</td><td>2, 13.3</td></tr>
<tr><td>at 9 years</td><td>0, 0</td><td>2, 28.6</td><td>2, 13.3</td></tr>
</table>

Supplemental Table 4: Extent of resection and clinical outcome in trigeminal nerve schwannomas

This table presents the patient and tumor characteristics, and postoperative course of tumors in patients with CN Vschwannomas. Data are stratified into the STR group and the TR group.

CN, cranial nerve; FU, follow up; TR, total removal; STR, subtotal removal

VII schwannoma		TR	STR	Total	*P* value
N, %		3, 75	1, 25	4	-
Age (range)		51.3, 32-74	59, 59	53.2, 32-74	1.00
Sex (n, %)	Male	1, 33.3	0, 0	1, 25	0.50
	Female	2, 66.7	1, 100	3, 75	
Tumor size at preoperation (mm, range)		30, 24-34	16, 16	26.5, 16-34	0.37
Tumor characteristics (n, %)	Solid	2, 66.6	0, 0	2, 50	0.29
	Cystic	1, 33.4	1, 100	2, 50	
Change of preoperative CN VII function after the operation (n, %)	Stabilized	1, 33.3	0, 0	1, 50	0.51
	Permanent deterioration	2, 66.6	1, 100	1, 50	
Tumor regrowth (n, %)		0, 0	1, 100	1, 25	0.05
Mean FU time (m, range)		51.6, 12-72	108, 108	65.7, 12-108	-
Improvement of CN VII function at 1,3,5,7,9 years (n, %)	at 1year	3, 100	1, 100	4, 100	-
	at 3years	2, 66.6	1, 100	3, 75	
	at 5 years	2, 66.6	1, 100	3, 75	
	at 7 years	0, 0	1, 100	1, 25	
	at 9 years	0, 0	1, 100	1, 25	

Supplemental Table 5: Extent of resection and clinical outcome in facial nerve schwannomas

This table presents the patient and tumor characteristics, and postoperative course of tumors in patients with CN VII schwannomas. Data are stratified into the STR group and the TR group.

CN, cranial nerve; FU, follow up; TR, total removal; STR, subtotal removal

LCN schwannoma		TR	STR	Total	*P* value
N, %		6, 50	6, 50	12	-
Age (range)		48.3, 43-62	45.8, 16-62	47.1, 16-62	0.75
Sex (n, %)	Male	4, 66.6	3, 50	7, 58.3	0.56
	Female	2, 33.3	3, 50	5, 41.7	
Tumor size at preoperation (mm, range)		26.8, 15-36	35.2, 19-49	31, 15-49	0.21
Tumor characteristics (n, %)	Solid	3, 50	2, 33.3	5, 41.7	0.56
	Cystic	3, 50	4, 66.7	7, 58.3	
Change of preoperative LCN function after the operation (n, %)	Stabilized	5, 83.3	6, 100	11, 88.9	0.30
	Permanent deterioration	1, 16.7	0, 0	1, 16.7	
Tumor regrowth (n, %)		0, 0	5, 83.3	5, 41.7	0.01
Mean FU time (m, range)		23.3, 13-72	69.6, 18-120	46.5, 13-120	-
Improvement of LCN function at 1,3,5,7,9 years (n, %)	at 1year	6, 100	6, 100	12, 100	-
	at 3years	2, 33.3	5, 83.3	7, 58.3	
	at 5 years	2, 33.3	4, 66.7	6, 50	
	at 7 years	0, 0	2, 33.3	2, 16.7	
	at 9 years	0, 0	1, 16.7	1, 8.2	

Supplemental Table 6: Extent of resection and clinical outcome in lower cranial nerve schwannomas

This table presents the patient and tumor characteristics, and postoperative course of tumors in patients with LCN schwannomas. Data are stratified into the STR group and the TR group.

FU, follow up; TR, gross total removal; STR, subtotal removal

XII schwannoma		TR	STR	Total
N, %		0, 0	3, 100	3, 100
Age (range)		-	51, 38-70	51, 38-70
Sex (n, %)	Male	-	2, 66.7	2, 66.7
	Female	-	1, 33.3	1, 33.3
Tumor size at preoperation (mm, range)		-	49, 31-63	49, 31-63
Tumor characteristics (n, %)	Solid	-	3, 100	3, 100
	Cystic	-	0, 0	0, 0
Change of preoperative LCN function after the operation (n, %)	Stabilized	-	3,100	3,100
	Permanent deterioration	-	0.0	0,0
Tumor regrowth (n, %)		-	2, 66.7	2, 66.7
Mean FU time (m, range)		-	87.6, 72-120	87.6, 72-120
Improvement of CN XII function at 1,3,5,7,9 years (n, %)	at 1year	-	3, 100	3, 100
	at 3years	-	3, 100	3, 100
	at 5 years	-	3, 100	3, 100
	at 7 years	-	1, 33.3	1, 33.3
	at 9 years	-	1, 33.3	1, 33.3

Supplemental Table 7: Extent of resection and clinical outcome in hypoglossal nerve schwannomas

This table presents the patient and tumor characteristics, and postoperative course of tumors in patients with CNXII schwannomas. Data are stratified into the STR group and the TR group.

CN, cranial nerve; FU, follow up; TR, total removal; STR, subtotal removal
